# The metal-nonoate Ni(SalPipNONO) inhibits *in vitro* tumor growth, invasiveness and angiogenesis

**DOI:** 10.18632/oncotarget.24350

**Published:** 2018-01-30

**Authors:** Valerio Ciccone, Martina Monti, Enrico Monzani, Luigi Casella, Lucia Morbidelli

**Affiliations:** ^1^ Department of Life Sciences, University of Siena, Siena, Italy; ^2^ Department of Molecular Medicine and Development, University of Siena, Siena, Italy; ^3^ Noxamet Ltd, Milan, Italy; ^4^ Department of Chemistry, University of Pavia, Pavia, Italy

**Keywords:** nitric oxide donor, lung cancer cells, apoptosis, angiogenesis, vascular endothelial growth factor

## Abstract

Nitric oxide (NO) exerts conflicting effect on tumor growth and progression, depending on its concentration. We aimed to characterize the anti-cancer activity of a new NO donor, Ni(SalPipNONO) belonging to the class of metal-nonoates, in epithelial derived tumor cells, finally exploring its antiangiogenic properties. Tumor epithelial cells were screened to evaluate the cytotoxic effect of Ni(SalPipNONO), which was able to inhibit cell proliferation in a dose dependent manner, being more effective than the commercial DETA/NO. The human lung carcinoma cells A549 were chosen as model to study the anti-cancer mechanisms exerted by the compound. In these cells, Ni(SalPipNONO) inhibited clonogenicity and cell invasion, while promoting apoptosis. The antitumor activity was partly due to NO-cGMP dependent pathway, contributing to reduced cell number and apoptosis, and partly to the salicylaldehyde moiety and reactive oxygen species (ROS) activated ERK1/2 signaling converging on p53 dependent caspase-3 cleavage. An additional contribution by downstream cycloxygenase-2 (COX-2) derived cyclopentenones may explain the tumor inhibitory activities. As NO has been described to affect tumor angiogenesis, we checked this activity both on tumor and endothelial cell co-cultures and in Matrigel *in vivo* assay. Our data document that Ni(SalPipNONO) was able to both reduce angiogenic factor expression by tumor cells acting on hypoxia inducible factor-1α (HIF-1 α) level, and endothelial cell functions related to angiogenesis. Collectively, these data confirm the potential use of NO donors and in particular Ni(SalPipNONO) acting through multiple mechanisms, as an agent to be further developed to be used alone or in combination with conventional therapy.

## INTRODUCTION

From the first studies on the effect of nitric oxide (NO) in cancer biology, this mediator emerged as a biphasic modulator, behaving both as an antineoplastic and proneoplastic stimulus [1]. The bimodal actions of NO can be explained through the duration of NO exposure, the cellular microenvironment, NO flux, tumor cell proliferation rate, occurrence of oxidizing and reducing processes [1].

The established biochemical/cellular events elicited by NO against tumor development are essentially inhibition of cell proliferation and proapoptotic events, and vascular effects including anti-angiogenesis. The proapoptotic events described in the literature relate to p53 upregulation and accumulation, degradation of antiapoptotic mediators, mitochondrial membrane permeability, and induction of cytochrome c release [2–4]. In cancer cells, NO released by NO donors or nitric oxide synthase (NOS) has been suggested to activate p53 *via* DNA damage by peroxynitrite (ONOO^–^) [5–7].

The use of NO donors can improve vascular flow, and anticancer drug delivery in hypoxic tissue, favoring the penetration of chemotherapy in tumor tissue and improving their cytotoxic effects [8–10]. Indeed, an increase in response to radiotherapy [10, 11] and chemotherapy [12, 13] has been reported.

Tumor tissue is characterized by low oxygen tension, a condition that promotes the activation and stabilization of hypoxia inducible factor-1α (HIF-1α) which, in turn, controls the transcription of vascular endothelial growth factor (VEGF), thus promoting angiogenesis, tumor growth and metastasis [14, 15]. NO has been reported to inhibit the expression of HIF-1α through the activation of HIF-1-prolyl hydroxylases and its proteasomal degradation [16–19]. NO, by reducing HIF-1α dependent VEGF levels, at the end can improve the delivery of antitumor drugs, through vascular normalization and reversion of the oncotic pressure gradient [20].

Recently, a new family of metal-nonoates has been developed [21] and characterized for their potential use in cardiovascular diseases, characterized by endothelial dysfunction, obtaining a vascular protective effect at nanomolar concentrations [22, 23]. Here, we have evaluated the antitumor activity of a member of this class, Ni(SalPipNONO), assessing the antitumor efficacy in two epithelial derived tumor cells, A549 and HT29, representative of lung and colon carcinoma, respectively. Ni(SalPipNONO) was characterized for different mechanisms related to tumor hallmarks as well as for its antiangiogenic effects on tumor and endothelial cells.

## RESULTS

### Antitumor effects and mechanisms of action of Ni(SalPipNONO)

**Figure 1:**
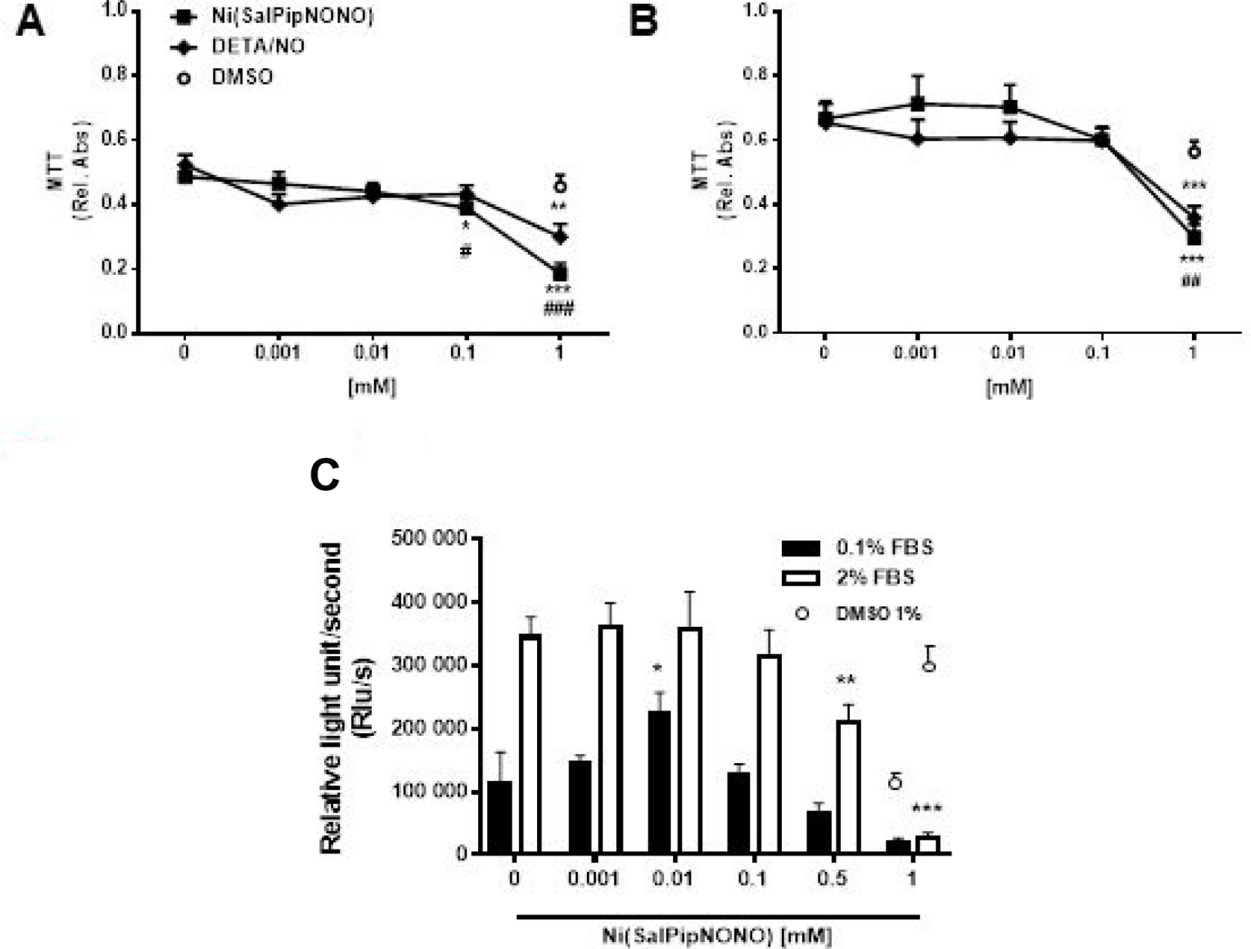
Ni(SalPipNONO) dose dependently inhibits tumor cell growth A549 cells were treated with increasing concentrations of NO donors (0.001–1 mM) in the presence of 0.1% (**A**) and 2% (**B**) serum and cell viability was evaluated by MTT after 72 h. Data are reported as relative absorbance ± SD (*n* = 3). Cell proliferation after 24 h was assessed by BrdU incorporation assay (**C**). Data are reported as luminescence ± SD (*n* = 3). The highest concentration of DMSO (1% v/v) used as vehicle was reported as control. ^*^p < 0.05, ^**^p < 0.01 and ^***^p < 0.001 vs untreated cells. ^#^*p* < 0.05, ^##^*p* < 0.01 and ^###^*p* < 0.001 Ni(SalPipNONO) vs DETA/NO.

**Figure 2:**
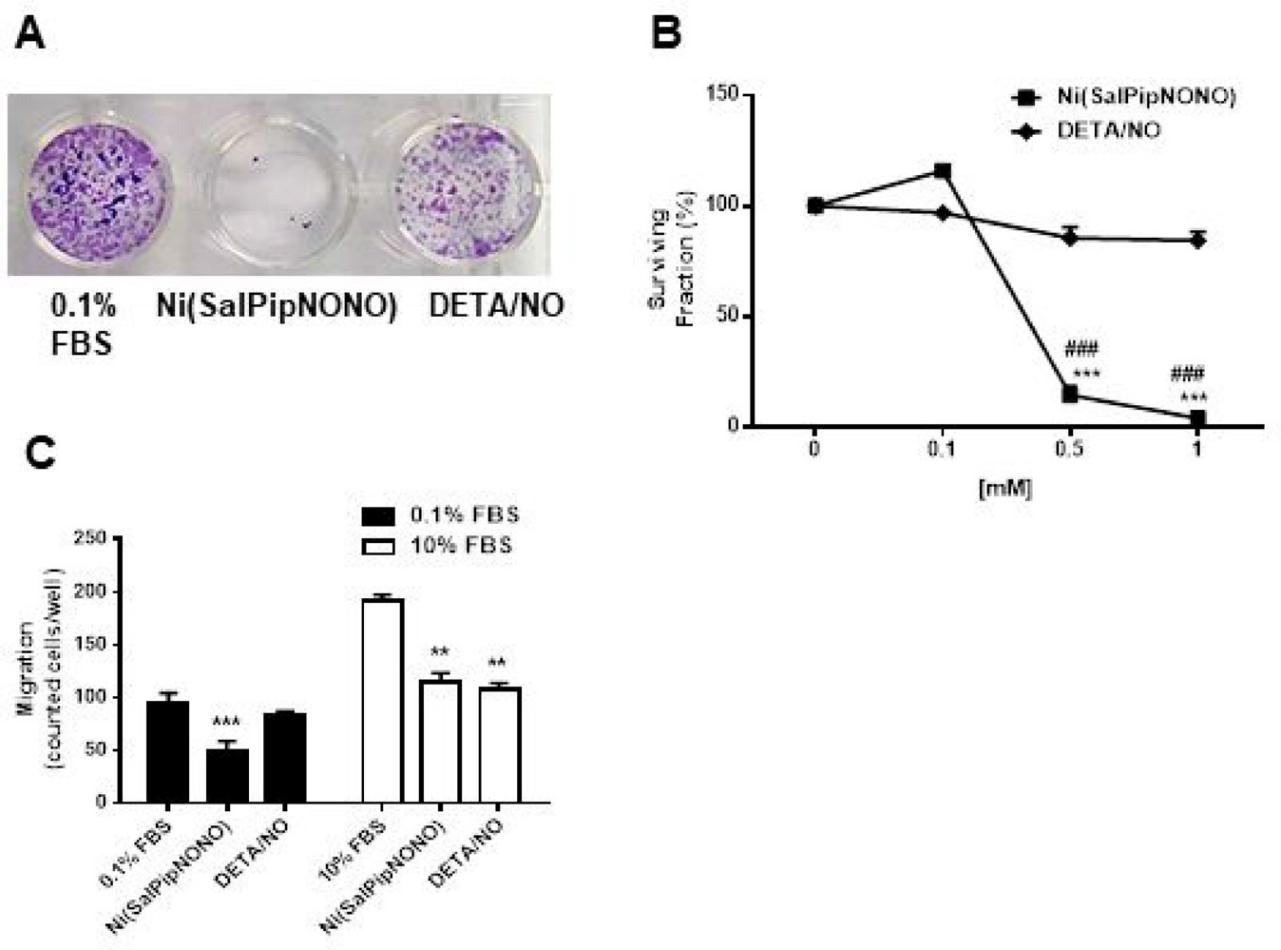
Ni(SalPipNONO) inhibits clonogenicity and invasiveness of A549 cells (**A**) A549 monolayers were treated with test NO donors for 48 h. After trypsinization cells were seeded at the density of 500 cells/well in 24 multi-well plates and let to form colonies for 10 days. Pictures of cultures were taken after fixation and staining. (**B**) The number of colonies formed by >30 cells were randomly counted/well and the graph represents the surviving fraction obtained giving 100% to untreated cells. (*n* = 3). ^***^p < 0.001 vs untreated cells and ^###^*p* < 0.001 Ni(SalPipNONO) vs DETA/NO. (**C**) Cell migration through a gelatin coated filter toward serum was assessed following incubation with Ni(SalPipNONO) or DETA/NO (0.5 mM) for 18 h. Data are reported as number of cells counted/well. (*n* = 3). ^**^p < 0.01 and ^***^p < 0.001 vs untreated cells.

To test the effect of novel NO donor, human lung carcinoma cells A549 cells were exposed for 72 h to Ni(SalPipNONO) and DETA/NO used in a wide range of concentrations (0.001–1 mM) and cell viability was assessed by the MTT assay. The experiment was performed in 0.1 and 2% FBS (Figure 1A and 1B). Ni(SalPipNONO), compared with equimolar concentrations of DETA/NO, was more effective in reducing cell number, in particular in the range 0.1–1 mM. The EC_50_ for Ni(SalPipNONO) were 0.26 and 0.37 mM in 0.1 and 2% serum, respectively. To assess the antiproliferative effect of the nonoate, BrdU incorporation assay were performed after 24 h of Ni(SalPipNONO) treatment in 0.1 and 2% FBS (Figure 2C). In both experimental conditions, the viability of A549 cells was less than 50% after exposure to 1 mM of NO donors. These experiments show that Ni(SalPipNONO) exerted its antiproliferative effects at doses near 0.5 mM, while at 1 mM it revealed a cytotoxic action.

Similar results were obtained with the human colon adenocarconma cells HT29 (Supplementary Figure 1A and 1B).

When tested on normal cells, namely HaCaT keratinocytes, Ni(SalPipNONO) exerted antiproliferative action only on sparse cells exposed to 2% serum (Supplementary Figure 2A). Interestingly, when the nonoate was tested on confluent cells exposed to low serum concentration, a more physiological condition, it did not inhibit cell proliferation (Supplementary Figure 2B) until 1 mM of drug.

Next, the cellular and biochemical characterization of Ni(SalPipNONO) antitumor activity was performed in A549 cells, using 0.5 mM concentration of the NO donor and control molecules. This concentration corresponded to a significant antiproliferative effects (on proliferating tumor cells) which allowed to study biochemical effects.

Beside cytotoxic/antiproliferative activity, we have studied the capability of metal-nonoate to interfere with clonogenicity of A549 cells. Data showed that at 0.5 and 1 mM of Ni(SalPipNONO) the surviving fraction was reduced of >90% with respect to basal, while DETA/NO exerted a modest, non-significant, effect (Figure 2A and 2B).

It is well known that metastasis of cancer cells involves cell invasiveness [24]. By using the Boyden chamber and gelatin coated filters, we have investigated the activity of NO donors on cell migration. As shown in Figure 2C, the test has been carried out in the presence of low and high concentration of an unspecific chemoattractant agent, i.e. serum. In both conditions, after 18 h of exposure to 0.5 mM Ni(SalPipNONO), we could detect halving of cells migrated across filter toward the lower chamber. The inhibitory effect of DETA/NO was evident only when cells were stimulated by a serum gradient.

From all these data, the metal-nonoate Ni(SalPipNONO) at sub-millimolar concentration showed an antiproliferative effect on lung cancer cells, accompanied by reduced clonogenicity and invasiveness.

We then evaluated the mechanisms responsible for the tumor inhibitory effects.

**Figure 3:**
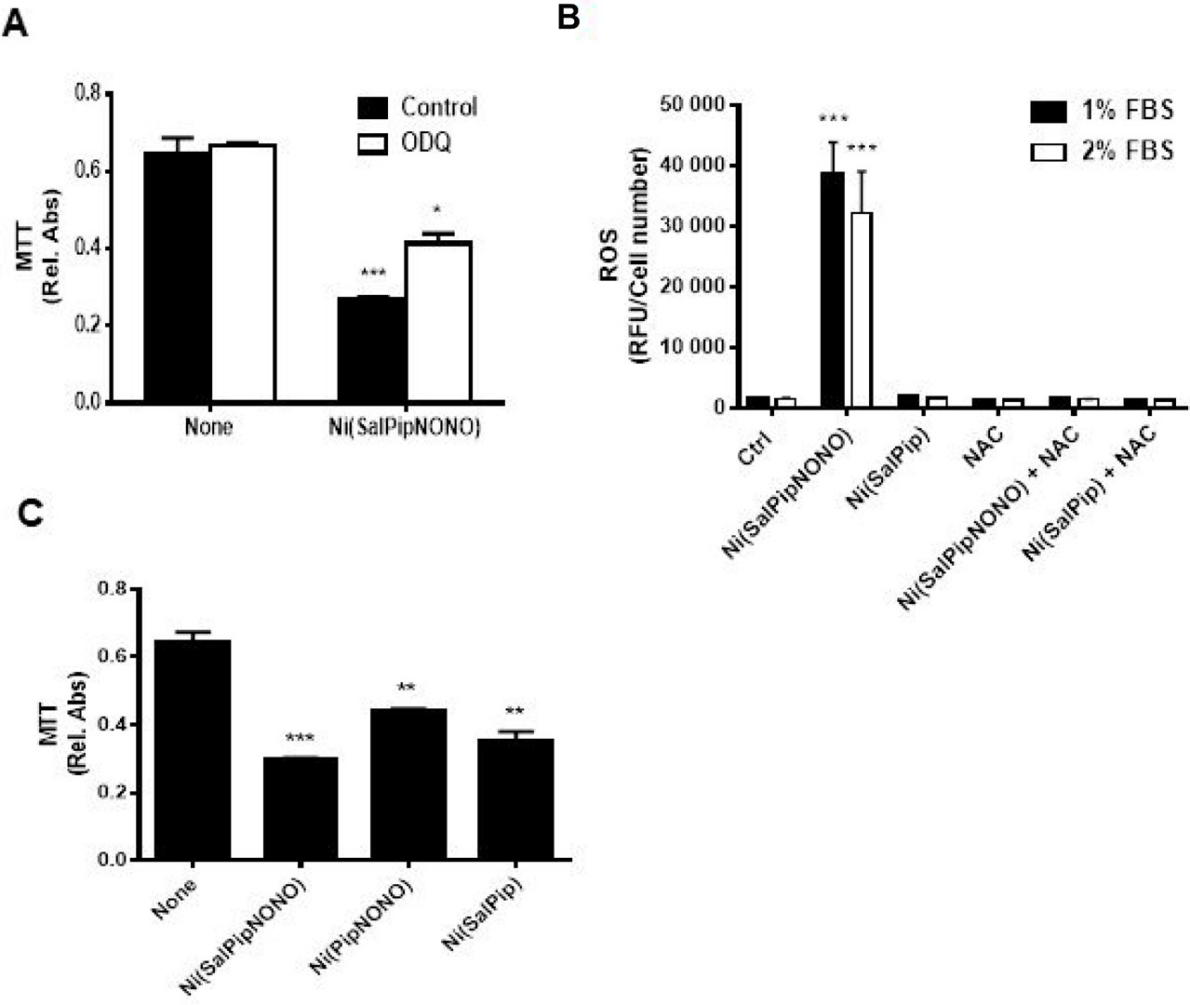
Molecular mechanisms responsible for cytotoxicity (**A**) sGC/cGMP involvement was evaluated by the use of 10 µM ODQ (30 min pretreatment) in survival assay of A549 cells treated with 0.5 mM Ni(SalPipNONO). Cell survival was evaluated by MTT. ^*^p < 0.05, ^***^p < 0.001 vs untreated cells. (**B**) Oxidative stress involvement was assessed by measuring ROS levels in A549 cells treated with Ni(SalPipNONO) and Ni(SalPip)Cl in the presence of different serum concentrations. NAC pretreatment (5 mM, 30 min) was used as a ROS scavenger. ^***^p < 0.001 vs untreated cells. (**C**) The contribution of NONO or salicylaldehyde moiety was evaluated by testing the cytotoxicity of molecules devoid of the NONO or salicylaldehyde group (each at 0.5 mM). Data are reported as relative absorbance of the MTT assay after 72 h incubation. ^**^p < 0.01 and ^***^p < 0.001 vs untreated cells.

First, we evaluated the involvement of the classical soluble guanylate cyclase (sGC)/cGMP pathway by the use of ODQ. The preincubation with ODQ (10 µM, 30 min before Ni(SalPipNONO) only partially reverted the NO donor cytotoxic effect (Figure 3A), suggesting that other pathways or chemical components of the molecule could be responsible of the antitumor actions. Oxidative stress and ROS production have been described to contribute to the cytotoxic effect of high doses on NO, due to its radical nature [6, 7]. We measured ROS levels in A549 cells stimulated with the metal-nonoate in different serum concentrations by means of the fluorophore DCFH2-DA and we found a significant burst in ROS production that was abolished by NAC pretreatment (Figure 3B).

Next, to evaluate the portion of molecule responsible of cytotoxic activity, we have tested different compounds with or without the NONO or the salicylaldehyde moiety at equimolar concentration. The compounds with salicylaldehyde group exerted the greater cytotoxic action (probably due to its metabolism in salicylic acid), with a synergetic effect with NONO group (Figure 3C). Ultimately, we can speculate that both the NONO group and salicylaldehyde moiety concur to the cytotoxic action. Since the compound devoid of NONO group did not create an oxidative environment in cells (Figure 3B), a double mechanism of cytotoxic action could be hypothesized: the NONO group induces oxidative stress, and salicylaldehyde exerts an additional mechanism.

**Figure 4:**
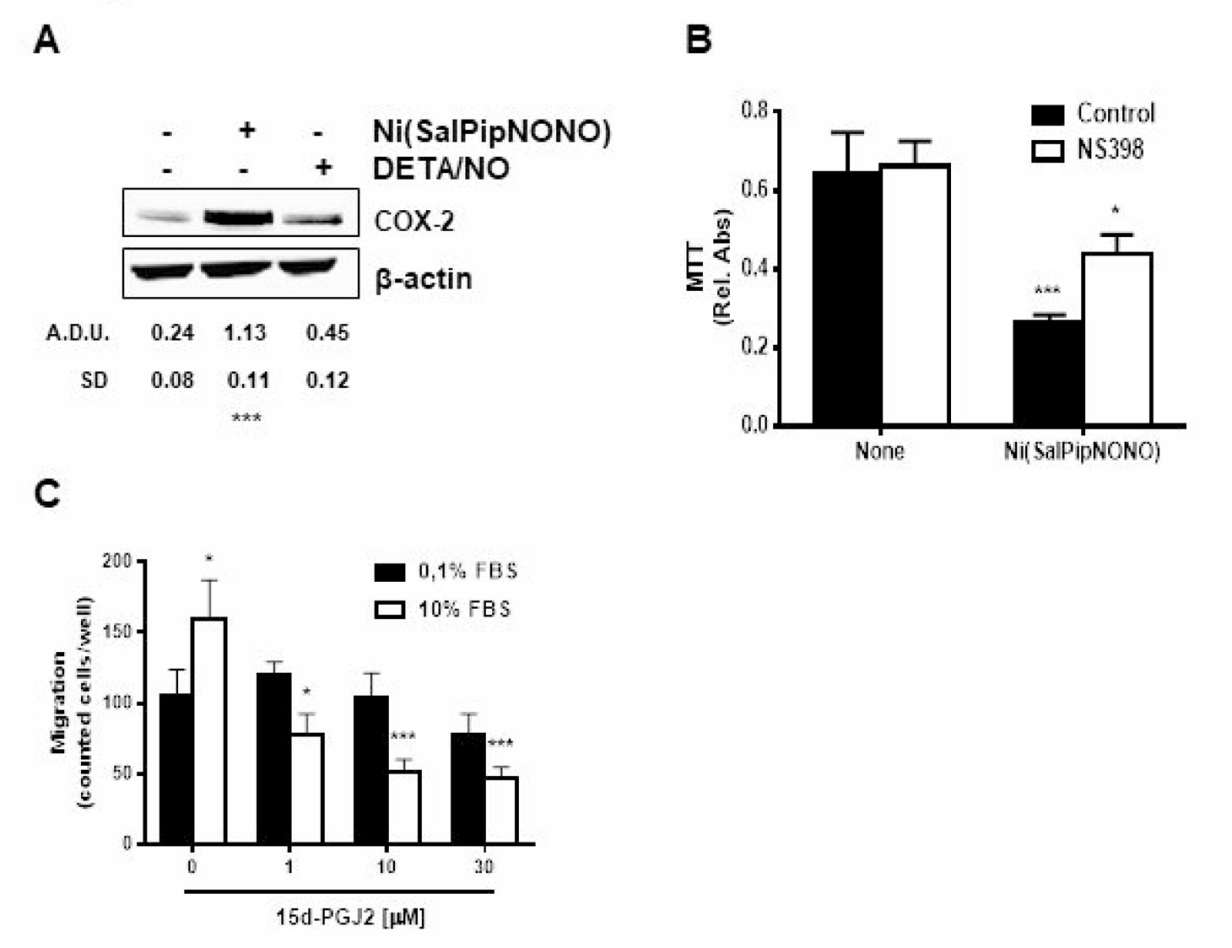
Ni(SalPipNONO) activates the COX-2 pathway (**A**) A549 cells were treated with Ni(SalPipNONO) or DETA/NO (0.5 mM) for 4 h. COX-2 expression was evaluated by western blot. ^***^p < 0.001 vs untreated cells. (**B**) The involvement of COX-2 activity on NO donor induced cytotoxicity was evaluated by means of NS398 (10 μM, 30 min pretreatment) incubation and MTT assay. ^*^p < 0.05 and ^***^p < 0.001 vs untreated cells. (**C**) Tumor cell migration toward serum was evaluated in cells treated with increasing concentrations of 15d-PGJ_2_. ^*^p < 0.05, ^**^p < 0.01 and ^***^p < 0.001 vs untreated cells.

Since COX-2 derived prostanoids can influence tumor development, and inflammation is one of the tumor hallmarks [24], we have evaluated the activity of Ni(SalPipNONO) on COX-2 protein levels. As reported in Figure 4A, COX-2 was upregulated by 0.5 mM concentration of the metal-nonoate, but not by DETA/NO. From a mechanistic point of view, the inhibition of COX-2 activity by NS398 partially reverted the metal-nonoate cytotoxic effect (Figure 4B), hypothesising that inhibitory prostanoids could contribute to antitumor activity. Indeed, cyclopentenones as 15d-PGJ_2_ have been reported to inhibit cell growth and induce apoptosis in various tumors [25–29]. In our tumor model, when cells were exposed to exogenous 15d-PGJ_2_, a dose dependent inhibition of cell chemotaxis toward 10% serum was detected (Figure 4C), suggesting a contribution of endogenously processed inhibitory prostanoids.

**Figure 5:**
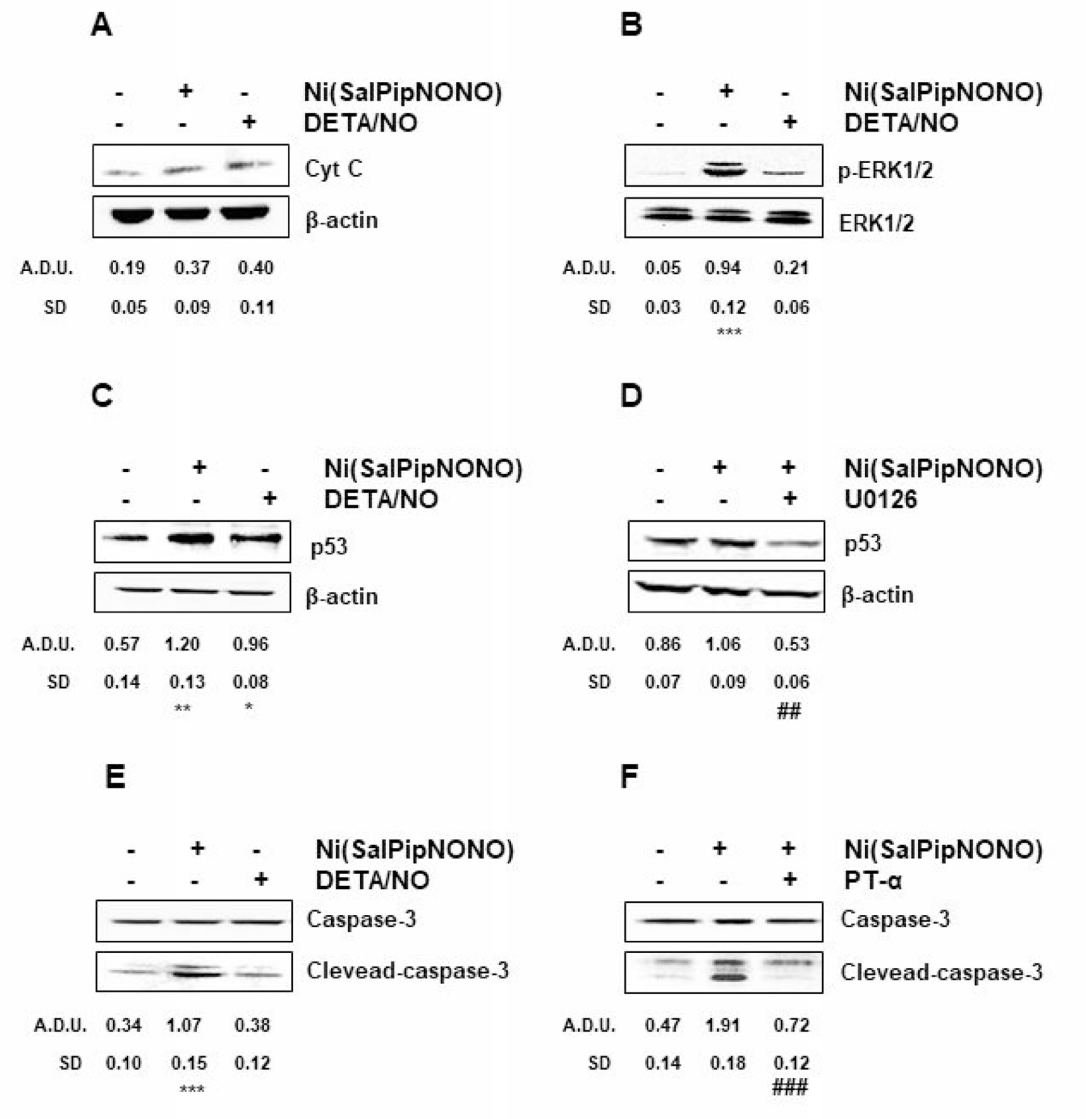
Ni(SalPipNONO) induces signaling related to apoptosis A549 cells are treated with Ni(SalPipNONO) or DETA/NO (0.5 mM) for various times ((**A**) 15 min; (**B**) 30 min; (**C**) and (**D**) 1 h; (**E**) and (**F**) 4 h). When inhibitors (U0126 or PT-α, each at 10 µM) are used, cells are pretreated for 30 min before stimulation with NO donors. The key signals are evaluated by western blot, using beta actin, total ERK1/2 or total caspase-3 as controls, when appropriate. Blots are representative of at least 3 with overlapping results. ^*^p < 0.05, ^**^p < 0.01 and ^***^p < 0.001 vs untreated cells. ^##^*p* < 0.01 and ^###^*p* < 0.001 vs Ni(SalPipNONO) alone.

The capability of NO to induce apoptosis is widely reported in literature [1, 2, 5, 6], thus to understand the mechanisms of cell number reduction promoted by Ni(SalPipNONO), the expression of apoptotic markers was evaluated. Exposure of cells to Ni(SalPipNONO) (0.5 mM, 15 min) doubled cytochrome c levels (Figure 5A), maybe through the reactive species generated by NO (like peroxynitrite and ROS) that cause opening of the permeability transition pore of mitochondria [2, 30]. Moreover, it is known that ROS activate ERK1/2 [31] and in A549 cells Ni(SalPipNONO), but not DETA/NO, strongly increased pERK1/2 after 30 min of incubation (Figure 5B). ERK1/2 on its turn upregulates p53 [32–34] and in our experiments p53 maximum level was obtained after 60 min of incubation with the metal-nonoate (Figure 5C). Indeed, the preincubation of the cells with MEK inhibitor U0126 interfered with p53 upregulation (Figure 5D).

The ultimate event downstream to cytochrome c release is caspase 3 activation that leads to DNA cleavage and apoptosis [35]. Our data show a maximum production (triple respect basal) of cleaved-caspase 3 after 4 h of exposure with Ni(SalPipNONO) (Figure 5E), an effect which was completely abolished by the preincubation with the p53 inhibitor PT-α (Figure 5F).

From all these data it results that Ni(SalPipNONO) is able to impair cell migration and reduce cell number and survival by activating the apoptosis pathway that passes through ROS production and COX-2 cyclopentenone activation, beside the partial involvement of the sGC/cGMP conventional signaling.

### Antiangiogenic activity by Ni(SalPipNONO) on tumor and endothelial cells

**Figure 6:**
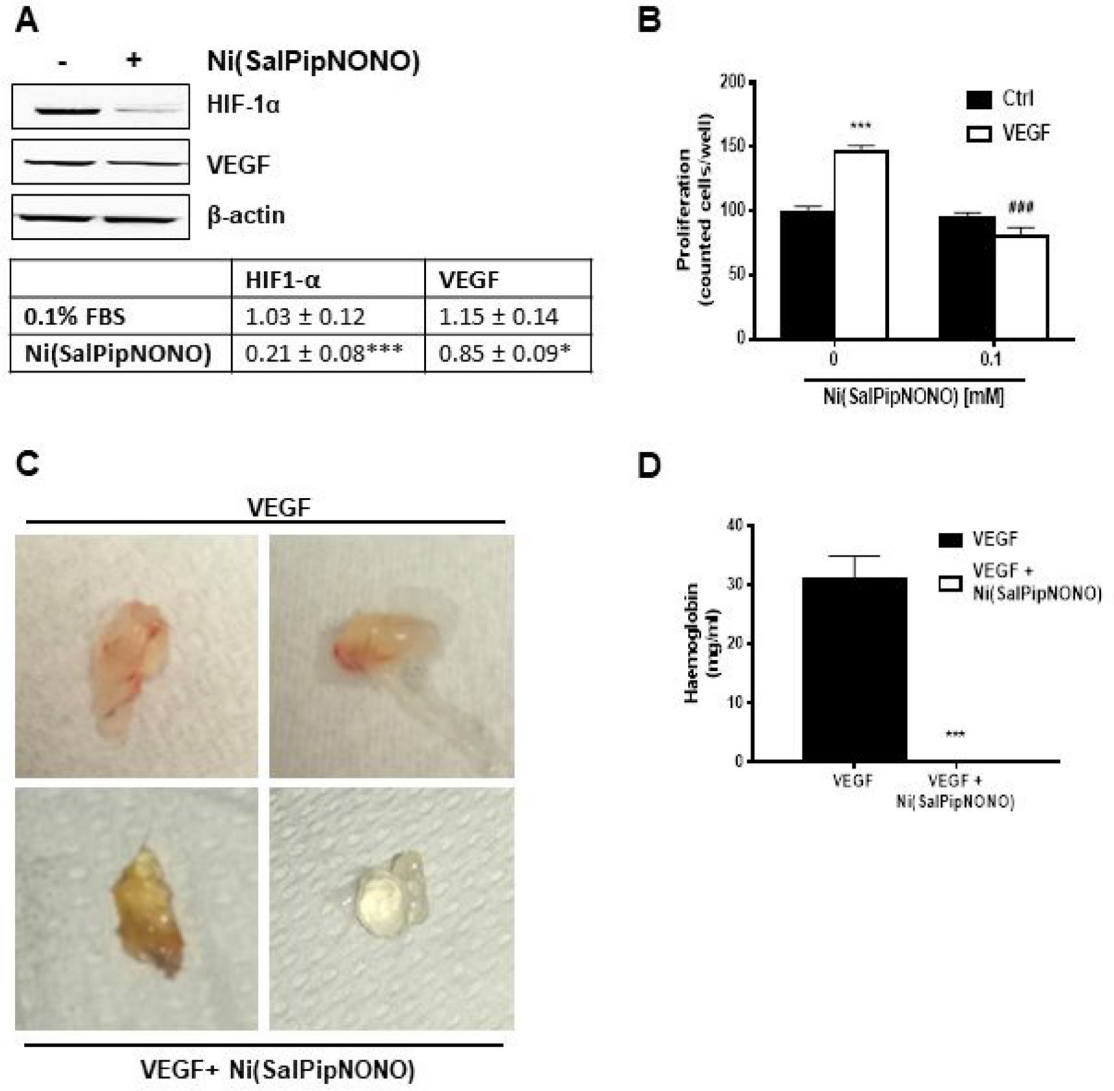
Ni(SalPipNONO) has antiangiogenic activities (**A**) A549 cells were treated with Ni(SalPipNONO) 0.5 mM for 24 h and HIF-1α and VEGF protein expression was evaluated by western blot. ^*^p < 0.05 and ^***^p < 0.001 vs untreated cells. (**B**) Direct antiagiogenic activity of Ni(SalPipNONO) on cultured endothelial cells. HUVEC were treated with VEGF (20 ng/ml) in the absence/presence of Ni(SalPipNONO) 0.1 mM. The number of cells was counted after 48 h following cell fixation and staining. ^***^p < 0.001 vs untreated cells. ^###^*p* < 0.01 vs VEGF alone. (**C**) Ni(SalPipNONO) exerts antiangionic activity *in vivo*. Mice were subcutaneously implanted with Matrigel containing VEGF (300 ng) in the absence or presence of 0.5 mM Ni(SalPipNONO). Plugs were removed after 10 days. Pictures are representative plugs out of 3, while the graph (**D**) reports hemoglobin content quantified by Drabkin reagent (*n* = 3 plugs). ^***^p < 0.001 vs VEGF alone.

HIF-1α expression and proangiogenic factor levels are correlated with an increased risk of mortality in several types of carcinoma [14]. In A549 cells, we could find HIF-1α detectable levels already in normoxic condition (Figure 6A). When A549 cells were treated for 24 h with Ni(SalPipNONO), there was a substantial decrease of HIF-1α expression and a consequent reduction of VEGF levels (Figure 6A). These results demonstrate that the metal-nonoate reduces the angiogenetic signals in tumor cells.

We then assessed whether the antiangiogenic property was directly induced in endothelial cells. HUVEC were exposed to VEGF (20 ng/ml) in the presence of Ni(SalPipNONO). Interestingly, at 0.1 mM the NO donor completely abolished VEGF induced proliferation (Figure 6B). The antiangiogenic activity of Ni(SalPipNONO) was evident also in an *in vivo* angiogenesis assay as the subcutaneous Matrigel plug implant. After 10 days, the presence of metal-nonoate abolished the vascularization produced by VEGF, as documented by representative pictures and hemoglobin content in the plugs (Figure 6C and 6D).

**Figure 7:**
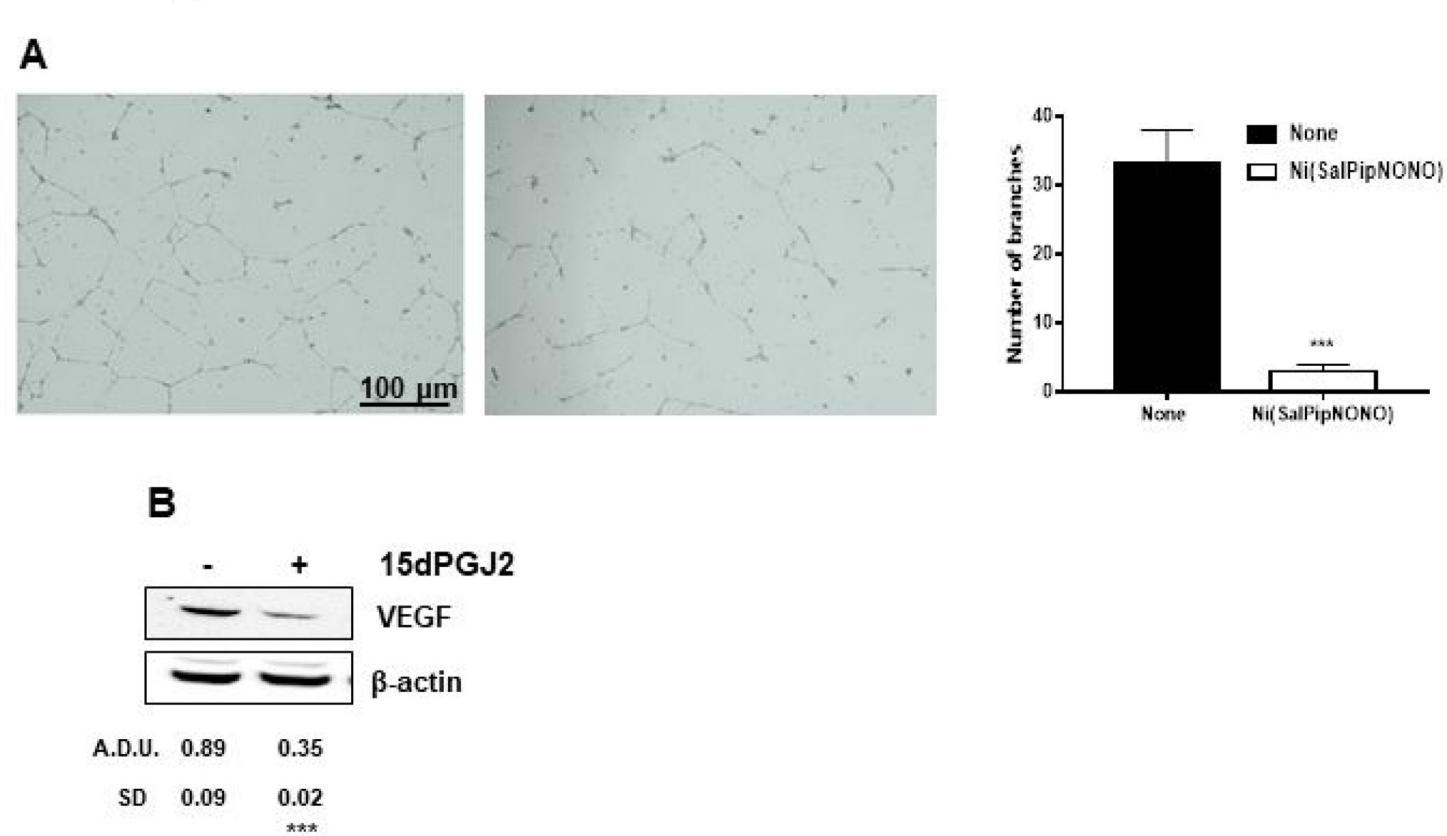
Ni(SalPipNONO) exerts antiangiogenic activity in a co-culture system (**A**) A549 cells were seeded on transwell inserts and treated or not with 0.5 mM Ni(SalPipNONO) for 54 h. Then the inserts (with tumor conditioned medium) were transferred on multiplates with HUVEC seeded on top of a Matrigel layer. Incubation continued with further 18 h. Pictures of HUVEC network were taken [a. medium conditioned by not treated A549; b. medium conditioned by A549 treated with Ni(SalPipNONO)] and are representative of 3 experiments. (**B**) Expression of VEGF by tumor cells exposed for 8 h to 10 µM 15d-PGJ_2_. The blot is representative of 3 with similar results. ^***^p < 0.001 vs untreated cells.

Co-culture experiments with tumor and endothelial cells were set up to strengthen the above results. Endothelial cell organization on Matrigel layers was evaluated in the presence of tumor cells grown on transwell inserts and treated or not with Ni(SalPipNONO). In the presence of untreated A549, in 18 h HUVEC formed net-like structures, which were impaired when tumor cells were treated with the NO donor (Figure 7A).Within the same time of incubation, a reduction in VEGF expression was observed after exposure of tumor cells to exogenous 15d-PGJ_2_ (Figure 7B).

These data in the whole demonstrate that the antiangiogenic activity of Ni(SalPipNONO) is both direct on endothelial cells and indirect on tumor cells which were not able to upregulate VEGF, probably through a COX-2/cyclopentenone dependent mechanism.

## DISCUSSION

Here we describe for the first time the antitumor efficacy of novel metal-nonoates, in particular Ni(SalPipNONO) on A549 tumor cells, characterizing the cellular and biochemical profile of the NO donor. We have found the cytotoxic activity of Ni(SalPipNONO) at doses higher than 0.1 mM, accompanied by inhibition of tumor clonogenicity and invasiveness, hallmarks typical of malignant tumors. From a mechanistic point of view, multiple pathways are responsible for the antitumor and proapoptotic events: sGC/cGMP activation; ROS production/p-ERK1/2 and cytochrome c/p53 pathway; COX-2/cyclopentenone contribution.

The literature reports the proapoptotic effect by various NO donors in tumors, contributing to radio-, chemosensibilitazion and photodynamic therapy [36–41]. Nitroglycerin (NTG) treatment was reported to significantly increase p53 phosphorylation at serine 15, and to enhance chemosensitivity to cisplatin in animal model of pathology [42]. Additional mechanisms, demonstrated following DETA/NO treatment, were S-nitrosylation and nuclear accumulation of p53 and start of intrinsic and extrinsic apoptotic pathway [43].

The influence of the conventional sGC/cGMP pathway is only marginal, since the block of the cascade with ODQ only partially reverted the cytotoxic effect of the metal-nonoate.

Here we demonstrate that Ni(SalPipNONO) promotes ERK1/2 phosphorylation, possibly through NO associated-ROS production. NO-induced oxidative stress influences MAPK dependent p53 upregulation and activation ultimately leading to caspase-3 cleavage. This effect is typical of Ni(SalPipNONO), since DETA/NO has lower capability to activate the MAPK pathway and apoptosis signalling and the compound devoid of the NONO group does not elicit an oxidative burst.

It is known that ROS mediated activation of ERK1/2 is able to activate the apoptotic pathway by antitumor agents [31]. ERK is part of the MAPK superfamily, and is well known for its ability to control cell survival in response to external stimuli [33, 44]. Several reports have found more complex roles for ERK pathway, in which the increase of ERK activity might promote apoptosis in specific environments. Protein kinase pathways such as the MAPK pathway are major oxidative stress-sensitive pathways in most cell types [45]. In particular, ERK is selectively activated in neuronal and renal epithelial cells upon exposure to oxidative stress and toxic agents such as cisplatin, and inhibition of the ERK pathway has been reported to block apoptosis [46]. Several studies have reported that curcumin potentiates ROS-dependent ERK activation and lethality in irradiated human cervical tumor cells [47] and that cisplatin-induced ERK activation is partly mediated through ROS generation [48]. In the current study, nonoate treatment induces a ROS burst and significantly increases ERK1/2 phosphorylation in A549 cancer cells. These results seem to be consistent with several earlier studies, in which increased ERK activity by reactive nitrogen species was linked to the induction of cell death [49].

The inhibitor of p53 PT-α blocked the apoptosome-mediated processing and activation of caspase-9 and -3 without interfering with the activation of mitochondria [50]. Our data show that apoptosis occurs via a p53-dependent mechanism that takes place upstream of mitochondria and involves cytochrome c release. Indeed, it is known that the reactive species generated by high levels of NO (both peroxynitrite and ROS) cause opening of the permeability transition pore of mitochondria [2, 30].

We have then evaluated the contribution of endogenous prostanoid pathway in the anti-tumor and antiangiogenic effect of the metal-nonoate.Among the COX-2 derived prostanoids, the pro-inflammatory PGE_2_ has a predominant role in promoting tumor growth [51]. However, both LOX-and COX-derived products can act as endogenous ligands of anti-proliferative and anti-tumorigenic receptor(s), as PPAR-γ. The cyclopentenone prostaglandins PGA_2_, PGA_1_, and PGJ_2_are formed by dehydration within the cyclopentane ring of PGE_2_, PGE_1_, and PGD_2_. PGJ_2_ is metabolized further to yield D12-PGJ_2_ and 15-deoxy-D12,14-PGJ_2_ (15d-PGJ_2_). Various compounds within the cyclopentenone prostaglandin family possess potent anti-inflammatory, anti-neoplastic, and anti-viral activity. Among cyclopentenones, 15d-PGJ_2_ has attracted our attention.15d-PGJ_2_, given exogenously, reproduces the antitumor activity of the metal-nonoate. 15d-PGJ_2_has been show to inhibit angiogenesis via suppression of pro-inflammatory enzymes and cytokines, even if stimulatory functions on tumor angiogenesis have been reported [52]. In human umbilical vein endothelial cells 15d-PGJ_2_ induced apoptosis via PPAR γ-dependent mechanisms [53].

The cyclopentenone involvement leads us to investigate the mechanisms related to the antiangiogenic activity of the metal-nonoate. Our *in vitro* and *in vivo* results document the antiangiogenic activity of Ni(SalPipNONO). Interestingly, the antiangiogenic activity of Ni(SalPipNONO) is both direct on endothelial cells and indirect on tumor cells via upregulation of VEGF. In the first ones it directly abrogates VEGF induced proliferative effect, while in tumor cells the nonoate inhibits HIF-1α dependent VEGF upregulation. An additional contribution of cyclopentenones as 15d-PGJ_2_ in preventing VEGF expression is here demonstrated.

A direct antiangiogenic activity by exogenous NO has been reported with other NO donors. *In vitro* assay with cultured endothelial cells revealed that the NO hybrid compound NCX-4016, significantly inhibited angiogenesis in a dose-dependent manner, with almost complete inhibition at a 100 μM concentration [54]. And recently, alteration of the expression of angiogenic genes has been described in hepatoma cells in response to NO donors, in particular an increase in thrombospondin-1 and tissue inhibitors of metalloprotease-1 [55], known inhibitors of angiogenesis and tumor cell migration.

In conclusion, these data confirm the potential use of NO donors and in particular Ni(SalPipNONO) acting through multiple mechanisms, as chemotherapeutic agents to be further developed in order to be used alone or in combination with anticancer conventional therapy. The results of a randomized phase II clinical trial have been reported, documenting the feasibility of the concurrent use of NTG with chemotherapy and radiotherapy in locally advanced non-small cell lung cancer to increase chemo- and radiosensitivity with an acceptable toxicity profile [56]. Interestingly, the overall survival was associated to reduced levels of circulating VEGF. Accordingly, a retrospective study suggested that application of NTG plus docetaxel and carboplatin in patients with operable lung adenocarcinoma increases the response with decreased expression of HIF-1α and VEGF [57], supporting an antiangiogenic activity.

## MATERIALS AND METHODS

### Drugs used

The metal-nonoates Ni(SalPipNONO), Ni(PipNONO)Cl and Ni(SalPip)Cl have been synthetized and characterized by Noxamet Ltd (Milan, Italy) as previously reported [21]. DETA/NO was from Sigma-Aldrich (St. Louis, MO, USA). All NO donors and control compounds were dissolved in DMSO (100 mM solution). Matrigel (growth factors and phenol red-free) was from Becton Dickinson (Waltham, MA, USA). VEGF was from R&D Systems (Minneapolis, MN, USA). The cyclopentenone prostaglandin 15-deoxy-D12,14-prostaglandin J2 (15d-PGJ_2_) was from Cayman Chemical (Ann Arbor, MI, USA).

The cyclic pifithrin-alpha (PT-α, a cell permeable and potent p53 transcriptional inhibitor) [50] was from Sigma Life Science (Milan, Italy). The MEK inhibitor U0126 (1,4-diamino-2,3-dicyano-1,4-bis(2-aminophenylthio)butadiene) and NS398, a cycloxygenase-2 (COX-2) inhibitor, were from Calbiochem (Milan, Italy). N-acetyl-L-cysteine (NAC) was from Sigma-Aldrich (St. Louis, MO, USA).

### Cell cultures

The human lung carcinoma A549 cells were obtained from European Collection of Cell Cultures (Salisbury, UK). HT-29, the human colorectal adenocarcinomacells, were obtained from the American Type Culture Collection. HaCaT immortalized human keratinocytes were chosen as human normal cell controls. HaCaT cells (passages 3–7) were acquired from Voden Medical (Meda, MB, Italy). Cells were maintained in DMEM (Sigma-Aldrich) supplemented with 10% fetal bovine serum (FBS) (Hyclone, Celbio, Milan, Italy) and 2 mM glutamine, 100 units penicillin and 0.1 mg/l streptomycin (Sigma Aldrich, St. Louis, MO, USA).

Human umbilical vein endothelial cells (HUVEC) were purchased from Promocell (Heidelberg, Germany) and were grown in endothelial growth medium (EGM-2), containing VEGF, R^3^-IGF-1, hEGF, hFGF, hydrocortisone, ascorbic acid, heparin and GA-1000 (Lonza, Basel, Switzerland), 10% FBS and 2 mM glutamine, 100 units/ml penicillin and 0.1 mg/ml streptomycin (Sigma Aldrich, St. Louis, MO, USA). Cells were split 1:3 twice a week, and used until passage 10. Cells were cultured at 37° C in 5% CO_2_.

### MTT assay

Tumor cell survivalwas quantified by MTT assay. 3 × 10^3^ cells were seeded in 96-multiwell plates in medium with 10% serum and after adherence were exposed to Ni(SalPipNONO) or DETA/NO (0.001 to 1 mM, 72 h) in 0.1% or 2% FBS. Where indicated, cells were pre-treated with 1H-[1,2,4]oxadiazolo[4,3-a]quinoxalin-1-one (ODQ) (10 µM) and NS398 (10 µM). Medium was removed and cells were incubated for 4 h with fresh medium in the presence of 1.2 mM 3-(4,5-dimethylthiazol-2-yl)-2,5-diphenyltetrazolium bromide (MTT). After solubilization in DMSO, absorbance was measured with a microplate absorbance reader (Infinite 200 Pro, Tecan Life Sciences, Switzerland) at 540 nm. Data are reported as 540 nm relative absorbance/well [58].

### BrdU incorporation assay

Cell proliferation was determined by 5-bromo-2′-deoxy-uridine (BrdU) incorporation using a chemioluminescence ELISA according to the manufacturer’s instructions (#11669915001 Roche Diagnostic S.p.A, Monza, Italy). To evaluate the effect on A549 cells, 3 × 10^3^ cells were seeded in 96-well plate. After adherence, cells were treated with Ni(SalPipNONO) (0.1 to 1 mM, 24 h) in presence of 0.1% e 2% FBS. To assay the effect on non-tumor cells on a condition of quiescence (to mimic a physiologic environment) 5 × 10^3^ cells were seeded in 96-well plate. After adherence, cells were treated with Ni(SalPipNONO) at 0.5 and 1 mM for 24 h in presence of 0.1% FBS. Finally, to test the antiproliferative action of drug on non-tumor cells, 3 × 10^3^ cells were seeded in 96-well plate. After adherence, cells were treated with Ni(SalPipNONO) at 0.5 and 1 mM for 24 h in presence of 2%. FBS. In all experimental conditions BrdU was added for the last 8 h of incubation. Then, cells were processed following manufacturer’s protocol. Chemiluminescence generated by BrdU labelled cells was measured using Infinite F200 Pro (Tecan Life Sciences, Switzerland).

### Clonogenic assay

The potential cytotoxic effect of NO donors was evaluated by the clonogenic assay [59]. A549 (2 × 10^5^ cells/well) were seeded in 6-well plates. After adherence, cells were treated with Ni(SalPipNONO) or DETA/NO (0.1 to 1 mM, 48 h). Then, cells were trypsinized, seeded in 24 multi-well plate at the density of 500 cells/well and incubated at 37° C for 10 days in medium with 1% serum. Colonies were fixed with methanol and stained with a solution of Cristal Violet in 10% methanol (Sigma Aldrich, St. Louis, MO, USA). Colonies formed by over 30 cells were counted and representative pictures were shown. Data are expressed as surviving fraction.

### Invasion assay

Cell invasion was performed by the Boyden chamber technique (Neuroprobe 48-well microchemotaxis chamber) (BiomapSnc, Agrate B.za, MI, Italy), with the filter coated with gelatin (Sigma Aldrich, St. Louis, MO, USA) [58, 59]. 1.25 × 10^4^ cells (A549) previously treated with Ni(SalPipNONO) or DETA/NO (0.5 mM, 18 h) or 15d-PGJ_2_ (1–30 µM) were added to the upper wells of the chamber. Lower wells contained 0.1% or 10% FBS as chemoattractant. After 4 h of incubation, cells were fixed and stained with Diff-Quik kit (Biomap Snc, Agrat B.za, MI, Italy). Migrated cells present in 5 fields/well were counted at 40× original magnification. Data are reported as the number of countedcells/well.

### ROS measurement

ROS levels were evaluated as previously reported [60]. A total of 1.5 × 10^3^ cells were seeded in 96-well plates and, after adherence, were treated with 0.5 mM Ni(SalPipNONO) or Ni(SalPip) in medium without phenol red and different serum concentrations. NAC (5 mM, 30 min pretreatment) was used as a ROS scavenger. DCFH2-DA (2,-7-dichlorodihydrofluorescein diacetate; Invitrogen, Milan, Italy) was added (10 μM, 30 min) and intracellular levels of ROS were evaluated with a microplate reader (excitation/emission 495/527; Infinite F200 Pro (TecanLifeSciences, Switzerland). The results are reported as relative fluorescence units (RFU) corrected for the cell number counted.

### Western blot

Sub-confluent A549 were seeded in 6 cm diameter Petri dishes. After adherence, cells were treated for the indicated times with Ni(SalPipNONO) (0.5 mM) and specific pathway inhibitors. Protein extraction and Western blot were performed as previously described [58, 61]. Electrophoresis (50 μg of protein/sample) was carried out in 4–12% Bis-Tris Gels (Life Technologies, Carlsbad, CA, USA). Proteins were then blotted onto nitrocellulose membranes, incubated overnight with primary antibodies [Anti-cytochrome c, anti-phospho-ERK1/2, anti-caspase- 3, anti-ERK antibodies from Cell Signaling (Celbio, Milan, Italy); anti-p53 antibody from Santa Cruz Biotechnology Inc (Dallas TX, USA); anti-COX-2 from Cayman Chemical (Ann Arbor, MI, USA); anti-HIF-1α from BD Biosciences (San Jose, CA, USA); anti-VEGF from Merck KGaA (Darmstadt, Germany)] and then detected by enhanced chemiluminescence system (Biorad, Hercules, CA, USA). Results were normalized to those obtained by using an antibody against beta actin from Merck KGaA (Darmstadt, Germany) or total ERK1/2 (Cell Signaling, Celbio, Milan, Italy), when indicated.

### Endothelial survival assay

Survival of endothelial cells (HUVEC) was evaluated following the protocol previously reported [61]. 1 × 10^3^ cells/well (of 96-well multiplates) were let to adhere in 10% serum for 3–4 h and then VEGF (20 ng/ml) in presence/absence of the NO donor was added in medium with 0.1% serum. After 2 days, cells were fixed, stained and randomly counted at 20 × original magnification in 5 fields. Data are reported as number of cell counted/well.

### *In vitro* co-culture assay

Tumor cells (3 × 10^4^ cells) were cultured on transwell inserts (12 mm diameter, polycarbonate membranes with 0.4 µm pores; Corning, Lowell, MA, USA) and treated for 24 h with 0.5 mM Ni(SalPipNONO). Then the inserts were transferred on top of endothelial cells plated on Matrigel (1.5 × 10^5^ cells in 12-well multiplate) for further 18 h of incubation. At the end of the experiment, endothelial cells were photographed and network formation on Matrigel was measured by means of the number of complete circles (Nikon Eclipse E400 and camera Nikon DS-5MC).

### *In vivo* matrigel angiogenesis assay

Investigation has been conducted in accordance with the ethical standards and according to the Declaration of Helsinki and the Italian law (Legislative Decree no.26, 4 March 2014), which acknowledges the European Directive 2010/63/UE, being approved by the authors’ institutional review board and the Italian Ministry of Health. All efforts were made to minimize the number of animals used and their suffering. *In vivo* Matrigel angiogenesis assay was performed as previously described [32]. C57 black mice (20–25 g) were kept in temperature- and humidity-controlled rooms (at 22° C) with lights on from 7 am to 7 pm, water and food available ad libitum. VEGF (300 ng) in presence/absence of Ni(SalPipNONO) (0.5 mM) was diluted in growth factor and phenol red-free Matrigel (Becton Dickinson, Franklin Lakes, NJ, USA) on ice. Mice were subcutaneously injected in the dorsal midline region with 0.4 ml of Matrigel. After 10 days, mice were euthanized and implants harvested. Plugs were re-suspended in 1 ml of Drabkin′s reagent (Sigma Aldrich, St. Louis, MO, USA) for 18 h on ice and hemoglobin concentration was determined by absorbance at 540 nm and compared with a standard curve (Sigma Aldrich, St. Louis, MO, USA).

### Statistical analysis

Data represent means ± SD of at least 3 determinations. Statistical analysis was performed by means of Student’s *t* test for unpaired data or by analysis of variance, followed by Bonferroni’s test for comparison among groups of data; *p* < 0.05 was considered statistically significant.

## SUPPLEMENTARY MATERIALS FIGURES



## Abbreviations

(COX-2): cycloxygenase-2
(15d-PGJ2): 15-deoxy-D12,14-prostaglandin J2
(DCFH2-DA): 2,-7-dichlorodihydrofluorescein diacetate
(FBS): fetal bovine serum
(HUVEC): human umbilical vein endothelial cells
(HIF-1 α): hypoxia inducible factor-1 α
(MTT): (3-(4,5-dimethylthiazol-2-yl)-2,5-diphenyltetrazolium bromide)
(NAC): N-acetyl-L-cysteine
(NO): nitric oxide
(NOS): nitric oxide synthase
(NTG): nitroglycerin
(ODQ): 1H-[1,2,4]oxadiazolo[4,3-a]quinoxalin-1-one
(PT-α): pifithrin-alpha
(ROS): reactive oxygen species
(RFU): relative fluorescence units
(sGC): soluble guanylate cyclase
(VEGF): vascular endothelial growth factor
