# Supplementary material for: The metal-nonoate Ni(SalPipNONO) inhibits in vitro tumor growth, invasiveness and angiogenesis
Source: Oncotarget. 2018 Jan 30;9(17):13353–65. doi: 10.18632/oncotarget.24350 (PMC5862583; doi:10.18632/oncotarget.24350)
Supplement: Supplementary file 1 [file oncotarget-09-13353-s001.pdf]

The metal-nonoate Ni(SalPipNONO) inhibits *in vitro* tumor growth, invasiveness and angiogenesis

SUPPLEMENTARY MATERIALS

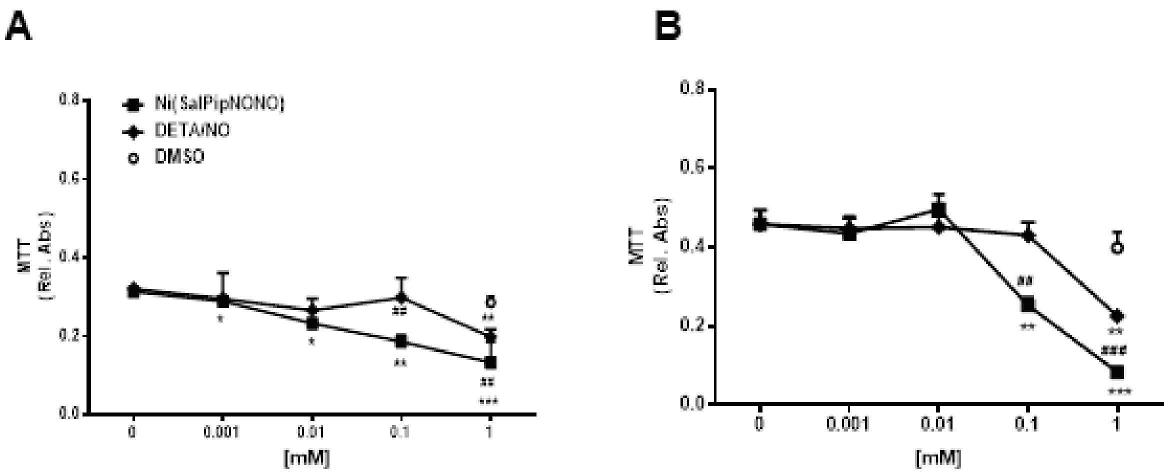

Supplementary Figure 1: Ni(SalPipNONO) dose dependently inhibits tumor cell growth. HT29 colon cancer cells were treated with increasing concentrations of NO donors (0.001–1 mM) in the presence of 0.1% (**A**) and 2% (**B**) serum and cell viability was evaluated by MTT after 72 h. Data are reported as relative absorbance ± SD (n = 3). The effect of 1% DMSO (vehicle of the NO donors) was tested as control. $^{*}p < 0.05$, $^{**}p < 0.01$ and $^{***}p < 0.001$ vs untreated cells. $^{\#}p < 0.05$, $^{\#\#}p < 0.01$ and $^{\#\#\#}p < 0.001$ Ni(SalPipNONo) vs DETA/NO.

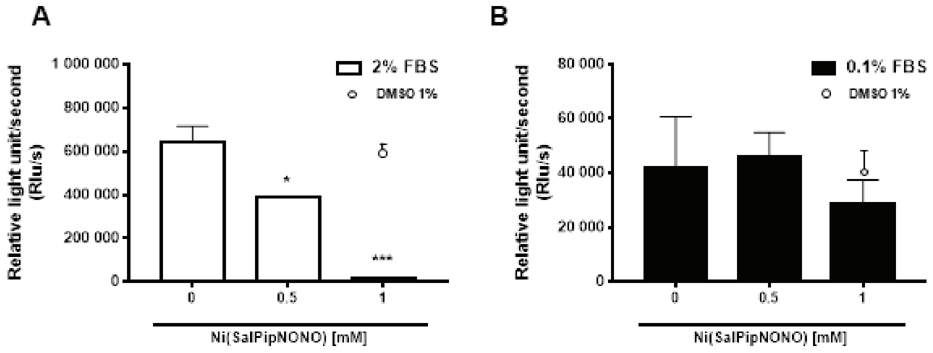

Supplementary Figure 2: Effect of Ni(SalPipNONO) on normal cells. The effect of Ni(SalPipNONO) was evaluated on human normal cells (HaCaT keratinocytes) by BrdU incorporation assay. Two different experimental conditions were assessed, one ((**A**) white columns, 2% FBS) with sparse and growing cells and the other ((**B**) black columns) with confluent cells exposed to low serum (0.1% FBS). (n = 3) $^{*}p < 0.05$ and $^{***}p < 0.001$ vs untreated cells.
